# Simulation Study of the Use of AlGaN/GaN Ultra-Thin-Barrier HEMTs with Hybrid Gates for Achieving a Wide Threshold Voltage Modulation Range

**DOI:** 10.3390/ma15020654

**Published:** 2022-01-15

**Authors:** Shouyi Wang, Qi Zhou, Kuangli Chen, Pengxiang Bai, Jinghai Wang, Liyang Zhu, Chunhua Zhou, Wei Gao, Bo Zhang

**Affiliations:** 1 State Key Laboratory of Electronic Thin Film and Integrated Devices, School of Electronic Science and Engineering, University of Electronic Science and Technology of China (UESTC), Chengdu 610054, China; wangshouyi@std.uestc.edu.cn (S.W.); chenkuangli86@std.uestc.edu.cn (K.C.); baipengxiang@std.uestc.edu.cn (P.B.); wangjinghai01@163.com (J.W.); uestc-zhuliyang@foxmail.com (L.Z.); gaoweidf@uestc.edu.cn (W.G.); zhangbo@uestc.edu.cn (B.Z.); 2 The Institute of Electronic and Information Engineering of UESTC in Guangdong, Dongguan 523808, China

**Keywords:** AlGaN/GaN HEMTs, threshold voltage modulation, ultra-thin barrier, normally off, enhancement-mode, p-GaN gate

## Abstract

In this work, novel hybrid gate Ultra-Thin-Barrier HEMTs (HG-UTB HEMTs) featuring a wide modulation range of threshold voltages (*V*_TH_) are proposed. The hybrid gate structure consists of a p-GaN gate part and a MIS-gate part. Due to the depletion effect assisted by the p-GaN gate part, the *V*_TH_ of HG-UTB HEMTs can be significantly increased. By tailoring the hole concentration of the p-GaN gate, the *V*_TH_ can be flexibly modulated from 1.63 V to 3.84 V. Moreover, the MIS-gate part enables the effective reduction in the electric field (E-field) peak at the drain-side edge of the p-GaN gate, which reduces the potential gate degradation originating from the high E-field in the p-GaN gate. Meanwhile, the HG-UTB HEMTs exhibit a maximum drain current as high as 701 mA/mm and correspond to an on-resistance of 10.1 Ω mm and a breakdown voltage of 610 V. The proposed HG-UTB HEMTs are a potential means to achieve normally off GaN HEMTs with a promising device performance and featuring a flexible V_TH_ modulation range, which is of great interest for versatile power applications.

## 1. Introduction

High-electron-mobility transistors (HEMTs) based on AlGaN/GaN heterostructures are promising for use in high-frequency and high-power applications due to their high breakdown voltage (BV) and polarization-induced two-dimensional electron gas (2DEG) at the interface of the AlGaN/GaN heterojunction [1]. However, the AlGaN/GaN HEMTs are inherently depletion-mode (D-mode) devices. In order to ensure power system security and simplify the gate drive circuit design, enhancement-mode (E-mode) devices with a sufficiently high V_TH_ are urgently wanted [2,3]. Several device structures have been reported to be able to realize normally off behavior. However, it is still quite difficult to effectively modulate the V_TH_ with a large range (e.g., >3 V) to accommodate various applications.

AlGaN/GaN metal-insulator (oxide) semiconductor (MOS/MIS) HEMTs with a positive V_TH_ can be achieved by a partially or fully recessed AlGaN barrier below the gate [4,5,6]. The 2DEG under the gate is effectively depleted and maintains a low gate leakage by introducing the gate dielectric. However, undesirable device performance degradation may occur due to the etching damage and presence of defect-related trap states. The presence of interface states introduced by etching in III-nitride compound semiconductor is one of the main factors that causes the instability of V_TH_ and decreases carrier mobility due to the scattering effect. The electron-trapping and detrapping during the device turn on and off may induce a dynamic shift in threshold voltage [7,8,9,10]. It is challenging to obtain a uniform V_TH_ in the manufacturing of MIS-HEMTs, particularly when the barrier thickness is less than several nanometers (e.g., <7 nm) [11,12]. Moreover, the presence of interface states may also lead to interface states-assisted gate leakage current.

Currently, the p-GaN gate HEMTs are commercialized to obtain E-mode operation, in which the p-GaN cap layer is inserted between the gate metal and AlGaN barrier to deplete the 2DEG beneath the gate [13]. Nevertheless, due to the low ionization rate of p-type dopants in GaN material, it is difficult to realize a high hole concentration to shift the V_TH_ even higher (e.g., >+3 V). Moreover, the time-dependent gate leakage was found to be consistent with the percolation of defects and gate reliability deterioration (e.g., the instability of the V_TH_ and even failure of the gate) normally originating from the E-field crowing effect in the p-GaN layer [14,15,16], which is critical in order for p-GaN gate HEMTs to deliver stable operation.

In recent years, ultra-thin barrier (UTB) HEMTs have obtained great attention. Benefitting from their unique advantage of being recess-free, the gate recess nonuniformity and the corresponding lattice damage and trap states can be effectively alleviated [17]. The as-grown thickness of the AlGaN barrier is less than 7 nm, which enables an intrinsic E-mode channel in the UTB AlGaN/GaN heterostructure. The high 2DEG density can be retained in the access region by depositing the LPCVD-SiN passivation due to the surface potential modulation effect and the presence of fixed positive charges at the SiN/AlGaN interface. The density of 3.26 × 10^13^ cm^−2^ for the positive fixed charges at the SiN/AlGaN interface has been confirmed [18,19]. The positive charges will enhance the 2DEG density in the access region, which can reduce the on-resistance of the device. In this manner, the intrinsic E-mode channel is reserved beneath the gate region, while the access region features a low on-resistance [20,21,22]. However, it has been reported that the V_TH_ of fabricated UTB HEMTs is less than 0.3 V (e.g., 0.27 V) [19]. In order to avoid a false turn on in power applications due to gate noise or the overshoot of gate control signal during the high speed switching, it is necessary to further increase the V_TH_. Therefore, to realize a high positive V_TH_ in UTB HEMTs is still challenging and demanding.

In this work, a hybrid gate structure featuring a p-GaN gate connected with a MIS- gate was introduced in the conventional UTB HEMTs to obtain a higher V_TH_. Owing to the effect of further depletion by the p-GaN cap, the range of V_TH_ modulation could be sufficiently increased by tailoring hole concentration in the p-GaN layer. The impact of the hole concentration, thickness, and Al-mole fraction of the AlGaN barrier on V_TH_ were investigated. Meanwhile, the MIS-gate effectively optimized the E-field distribution in the gate region. The E-field peak located at the drain-side edge of the p-GaN gate was well reduced, which was beneficial for obtaining a low gate leakage and improved the gate reliability. The proposed device structure and the concept of hybrid gate design are of great interest for fabricating the E-mode GaN power HEMTs with a flexible V_TH_ modulation range to accommodate different applications_._

## 2. Device Structure and Characteristics

### 2.1. Device Structure 

**Figure 1:**
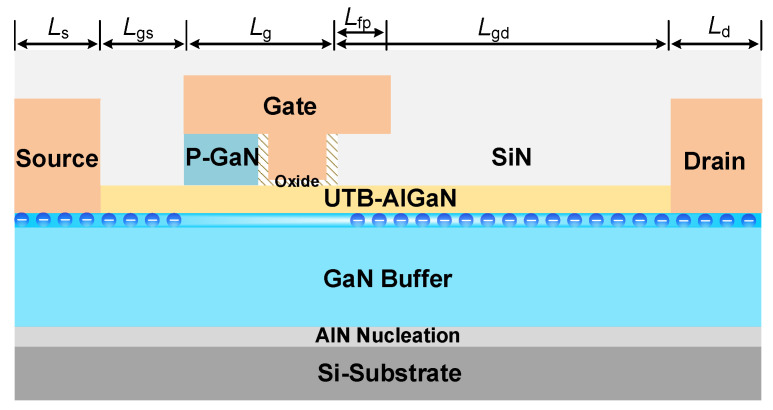
Schematic cross-sectional structure of the HG-UTB HEMTs.

The structure of the proposed HG-UTB HEMTs is shown in Figure 1. The hybrid gate structure consisting of a p-GaN/MIS-gate is introduced in the UTB HEMTs to break through the trade-off between V_TH_ modulation and increased channel resistance. Benefitting from the hybrid gate structure, the device could realize a wide range of threshold modulation and optimized E-field distribution near the gate.

**Figure 2:**
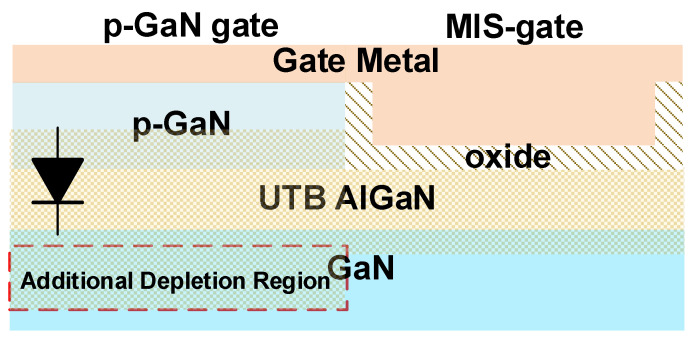
Depletion distribution under the gate region of HG-UTB HEMTs.

Due to the reduced polarization effect in the UTB HEMTs, the potential well at the heterointerface was raised above the Fermi level, which led to a native E-mode channel in the UTB heterojunction. The V_TH_ of conventional MIS-gate UTB HEMTs is mainly dominated by the thickness and Al-mole fraction of the AlGaN barrier. Even though the intrinsic E-mode channel resulted in a low positive V_TH_ (e.g., <0.3 V) [19], this was not high enough to deliver a safe operation in power electronic applications. The depletion region distribution under the hybrid gate region is shown in Figure 2. Owing to the sharp decrease in polarization intensity in UTB HEMTs, the depletion region mildly extended to the GaN buffer to achieve charge balance. It is noteworthy, that the p-GaN layer and 2DEG at the AlGaN/GaN heterojunction could be modeled as a p-i-n diode, the additional depletion region appeared and 2DEG density was dramatically decreased. Furthermore, the intensity of depletion under the gate region could be adjusted by tailoring the hole concentration in the p-GaN gate, which would enable the flexible threshold modulation.

Moreover, the drain-side MIS-gate part acted as a junction termination, which could transfer the E-field peak from the vulnerable p-GaN gate to the robust MIS-gate and thus reduce the gate leakage; this was also beneficial for gate reliability improvement.

To further study the device physics in depth, Sentaurus-TCAD was carried out. As a mainstream device simulation tool for micro-electronic devices, Sentaurus can be used to evaluate the device characteristics, understand the operation mechanism, and optimize the device structure. The critical device parameters used in the simulation are summarized in Table 1. The hole concentration in the p-GaN layer (*N*_p_) and the thickness of the AlGaN barrier layer (*T*_AlGaN_) are variables that can be used for studying their impact on the V_TH_ of HG-UTB HEMTs. Taking the low activation ratio of Mg dopant in GaN into account, a hole concentration of less than 5 × 10^17^ cm^−3^ was used in simulation, which is reported as experimentally achievable in the literature [14]. In HG-UTB HEMTs, gate metal formed Schottky contact with the p-GaN gate and the corresponding barrier height was 1.65 eV, which is comparable to previous reports in the p-GaN gate HEMTs [15,16].

### 2.2. V_TH_ Modulation Effects

**Figure 3:**
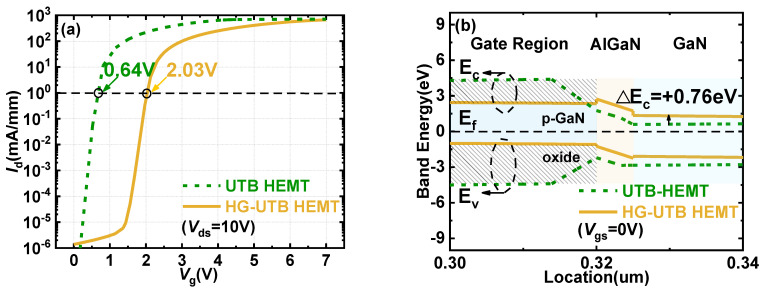
(**a**) Transfer characteristics of UTB HEMTs and HG−UTB HEMTs; (**b**) band energy profile at the left middle area of the gate region. The gate oxide and p−GaN for conventional MIS−gate UTB HEMTs and HG−UTB HEMTs, respectively, are shown.

To reveal the in-depth mechanism of HG-UTB HEMTs and evaluate the enhancement of the characteristics, conventional MIS-gate UTB HEMTs with the same device parameters (e.g., *L*_g_, *L*_gd_, *T*_AlGaN_, *X*_Al_, *T*_buffer_) were used for reference. As shown in Figure 3a, the V_TH_ was enhanced from 0.64 V in the conventional MIS-gate UTB HEMTs to 2.03 V in the proposed HG-UTB HEMTs with a comparable drain current. It is worth noting from Figure 3b that the energy band diagram at the AlGaN/GaN heterointerface was raised by 0.76 eV by the hybrid gate design. Hence, compared with the conventional MIS-gate UTB HEMTs, the 2DEG density dramatically decreased from 1.63 × 10^2^ cm^−2^ to 6.10 × 10^−5^ cm^−2^. Consequently, the HG-UTB HEMTs required a higher gate voltage to pull down the conduction band below the Fermi level to accumulate electrons and form the conduction channel, which led to much a higher V_TH_ being achieved in the HG-UTB HEMTs.

## 3. Results and Discussion

### 3.1. Impact of Hole Concentration and Polarization on V_TH_

In Figure 3b, it is shown that the elevation of 2DEG potential well under thermal equilibrium is proportional to the increase in V_TH_. The heterojunction polarization intensity (e.g., the thickness and the Al-mole fraction of the AlGaN barrier) and doping concentration in the p-GaN gate have a significant impact on the electron distribution under the gate and V_TH_. Thus, *X*_Al_, *T*_AlGaN_, and *N*_p_ are regarded as variables to study their impact on device characteristics (i.e., V_TH_). In order to enable normally off operation, the Al-mole fraction and barrier thickness of the UTB heterojunction were varied between 0.18 and 0.25 and 3 and 7 nm, respectively.

**Figure 4:**
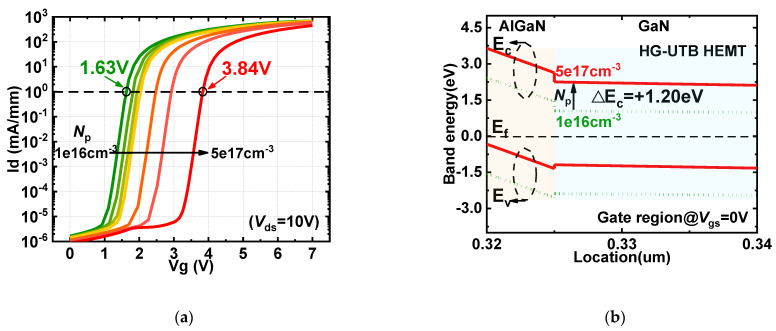
(**a**) *V*_TH_ modulation of HG−UTB HEMTs by varying *N*_p_; (**b**) energy band diagram under the gate region at AlGaN/GaN heterojunction with various *N*_p_.

Figure 4a shows the impact of hole concentration on the transfer characteristics when the barrier thickness and Al-mole fraction are 5 nm and 0.20, respectively. The HG-UTB HEMTs are capable of attaining V_TH_ increments over 2.2 V and maintaining a considerable conduction current with *N*_p_ varying from 1 × 10^16^ cm^−3^ to 5 × 10^17^ cm^−3^. Figure 4b shows the energy band diagram under the p-GaN gate with various hole concentrations. A P-GaN cap with a higher hole concentration (e.g., 5 × 10^17^ cm^−3^) can lift the potential well of the 2DEG channel higher (e.g., 1.20 eV). In this way, the 2DEG density beneath the p-GaN gate dramatically reduces from 1.78 × 10^0^ cm^−2^ to 4.10 × 10^−7^ cm^−2^. Compared with the p-GaN gate, the electron concentration under the MIS-gate is at least two orders of magnitude higher (e.g., 1.63 × 10^2^ cm^−2^). This explains why the hybrid gate structure can break the limitation of the *V*_TH_ modulation of conventional MIS-gate UTB HEMTs. 

**Figure 5:**
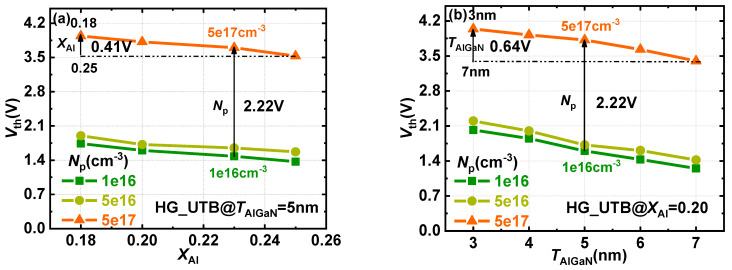
*V*_TH_ modulation of HG−UTB HEMTs with various *N*_p_ (**a**) and Al−mole fractions (*X*_Al_) (**b**) and thicknesses (*T*_AlGaN_).

Figure 5a,b, respectively, show the effect of the Al-mole fraction and barrier thickness on V_TH_ as a function of hole concentration in the p-GaN gate. As shown in Figure 5a, with the increase in *X*_Al_ from 0.18 to 0.25, the polarization intensity of the heterojunction increases and the V_TH_ decreases with a maximum variation range of 0.41 V. When the barrier thickness and hole concentration are 5 nm and 5 × 10^17^ cm^−3^, respectively, the V_TH_ reduces from 3.94 V to 3.53 V. Meanwhile, when the thickness and Al-mole fraction of the AlGaN barrier is 5 nm and 0.23, the *V*_TH_ increases from 1.48 V to 3.70 V, while the hole concentration increases from 1 × 10^16^ cm^−3^ to 5 × 10^17^ cm^−3^, respectively. Increasing the hole concentration can effectively raise up the energy band at the heterointerface and further reduce the electron concentration in the potential well, which is more effective in shifting the V_TH_ rather than varying the Al-mole fraction.

Similarly, increasing the thickness of the AlGaN barrier will increase the polarization charge density and enhance the 2DEG density, which may account for the downward trend of the V_TH_ shown in Figure 5b. When the Al-mole fraction and hole concentration are 0.20 and 5 × 10^17^ cm^−3^, respectively, the V_TH_ decreases from 4.04 V to 3.40 V with *T*_AlGaN_ being enhanced from 3 nm to 7 nm. When the Al-mole fraction and thickness of the barrier are 0.20 and 5 nm, the V_TH_ increases from 1.60 V to 3.82 V with *N*_p_ the increasing from 1 × 10^16^ cm^−3^ to 5 × 10^17^ cm^−3^. Compared with the decrease in the thickness of AlGaN barrier, the V_TH_ shift range is expanded by 2.5 times by tailoring the hole concentration.

Therefore, the introduction of the p-GaN gate in conventional MIS-gate UTB HEMTs can reconcile the contradiction between *V*_TH_ shift and drain current reduction, and the V_TH_ modulation range can be significantly enhanced by tailoring the hole concentration in the p-GaN layer.

### 3.2. E-Field Distribution in the Gate Region

From the discussion above, the p-GaN gate in the hybrid gate structure can effectively enhance the *V*_TH_ shift range with a negligible drain current reduction. On the other hand, the MIS-gate part of the hybrid gate structure in the HG-UTB HEMTs acts as a termination of the p-GaN gate, which transfers the E-field peak from the vulnerable p-GaN edge to the MIS-gate part. In this manner, the high peak E-field is sustained by the more robust MIS-gate for the improved gate reliability of the device, since the high E-field may induce undesirable electron trapping or hole injection effects in the p-GaN gate, which may lead to V_TH_ instability and even gate failure [14,15,16,23].

**Figure 6:**
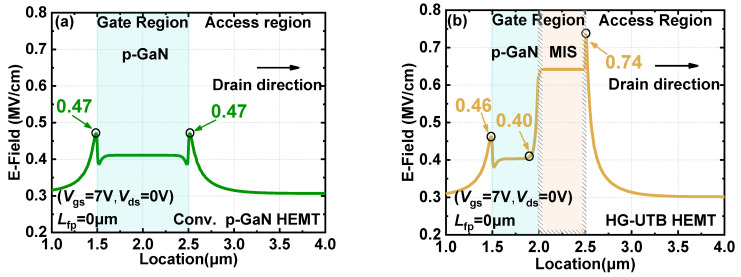
Distribution of the E-field beneath the gate region with gate bias of 7 V of (**a**) conventional p-GaN gate HEMTs and (**b**) HG-UTB HEMTs.

To study the performance of the E-field distribution of the hybrid gate structure, devices with a single p-GaN gate featuring identical device dimensions (e.g., *L*_g_, *L*_gd_, *T*_AlGaN_, *X*_Al_, *T*_buffer_) were utilized for comparison. The E-field profile along the 2DEG channel of the conventional p-GaN gate HEMTs and HG-UTB HEMTs in forward gate bias (e.g., +7 V) are shown in Figure 6a,b, respectively. Since cylindrical junction at the gate corner was formed, the E-field peaks appear at both gate edges (i.e., 1.5 µm and 2.5 µm). It can be seen that the E-field peak located at the p-GaN edge is 0.47 MV/cm in the conventional p-GaN gate HEMTs, as depicted in Figure 6a. In contrast, as for HG-UTB HEMTs shown in Figure 6b, the E-field peak at the drain-side gate edge of the p-GaN gate part is respectably suppressed while the E-field peak is removed to the drain-side edge of the MIS-gate part. Similarly, the E-field peak at the source-side edge is also reduced. In this manner, the high E-field at the gate region in the proposed HG-UTB HEMTs is sustained by the MIS-gate part, which may feature lower gate leakage due to the presence of the gate dielectric. Accordingly, the optimized E-field distribution in the gate area enables improved the gate reliability of the HG-UTB HEMTs.

**Figure 7:**
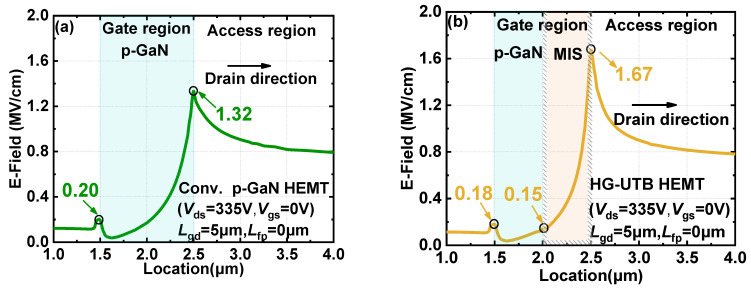
Distribution of E-field beneath the gate region in reverse blocking with a drain bias of 335V of (**a**) conventional p-GaN gate HEMTs and (**b**) HG-UTB HEMTs.

More importantly, the E-field strength was much higher in reverse blocking than that in forward conduction. Hence, the E-field distribution in reverse blocking is also investigated and compared in Figure 7 to validate the superiority of E-field distribution in the HG-UTB HEMTs by using the hybrid gate structure. The E-field profile along the channel of the conventional p-GaN gate HEMTs and the proposed HG-UTB HEMTs in off-state at 335 V, are shown in Figure 7a,b, respectively. Similarly, the E-field peak at the drain-side edge of the p-GaN gate was dramatically reduced by the hybrid gate structure from 1.32 MV/cm to 0.15 MV/cm. In Figure 7b, the high E-field of 1.67 MV/cm is sustained by the more robust MIS-gate. The MIS-gate part of the hybrid gate can promote the broadening of depletion along the drift region and thus improve the E-field crowing effect, which can reduce the possible gate leakage current and even alleviate the gate degradation that could be triggered in the Schottky type p-GaN gate. Moreover, the removal of high E-field away from the p-GaN gate, the typical electron trapping, and the hole injection observed in p-GaN gate can also be mitigated [23,24,25,26], which is beneficial for obtaining a more stable *V*_TH_ of the device.

### 3.3. Forward & Reverse Characteristics

In the UTB HEMTs, the LPCVD-SiN was used to restore the high-density 2DEG [19,20,21,22], while the fixed positive charge with a density of 3.26 × 10^13^ cm^−2^ at the SiN/AlGaN interface was proposed to be responsible for the 2DEG accumulation [19,27,28,29,30]. In this work, the fixed positive charge of 3.10 × 10^13^ cm^−2^ was used to imitate the LPCVD-SiN passivation effect in the access region to restore the 2DEG in the HG-UTB HEMTs.

**Figure 8:**
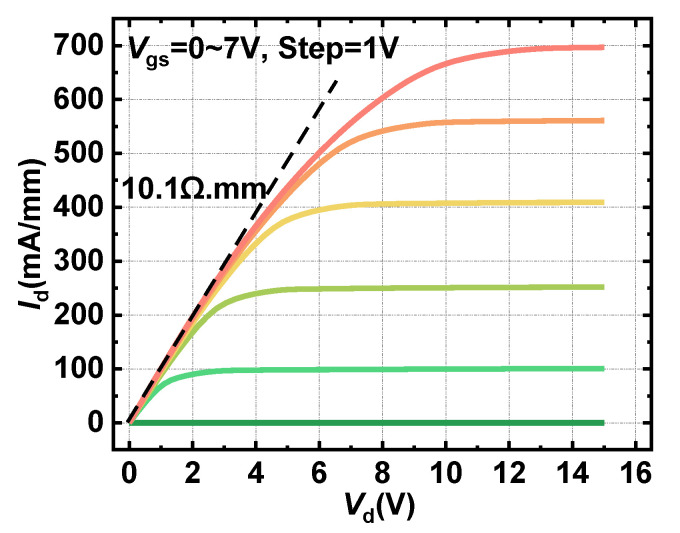
Output characteristics of the HG-UTB HEMTs.

Figure 8 illustrates the output characteristic of the proposed HG-UTB HEMTs. The maximum output current density up to 701 mA/mm was obtained, which yielded an on-resistance of 10.1 Ω mm.

**Figure 9:**
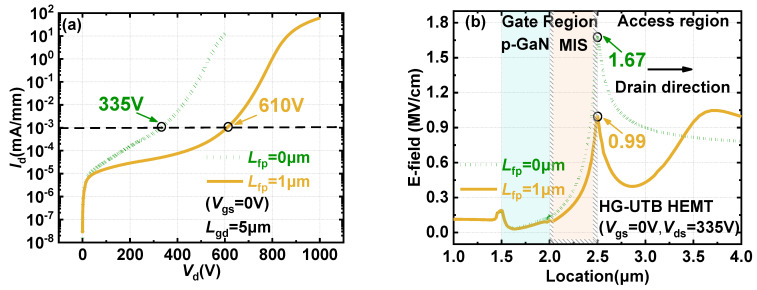
The impact of gate field plate on reverse characteristics of the HG−UTB HEMTs’ (**a**) breakdown performance and (**b**) E−field distribution along the channel.

Figure 9a shows the breakdown characteristics of HG-UTB HEMTs without a gate field plate and with a 1 μm gate field plate. The impact of a gate field plate on the E-field distribution along the channel at drain bias of 335 V is shown in Figure 9b. The field plate plays a role in enhancing the expansion of the depletion region in the block-state to obtain more uniform E-field distribution. The field plate reduced the E-field peak at the drain-side gate edge by 40% by introducing a new E-field peak with a comparable E-field strength at the gate field edge, which was beneficial to avoid the premature breakdown of the device. Therefore, the breakdown voltage of the device was significantly improved from 335 V to 610 V with a drift length *L*_gd_ of 5 µm.

## 4. Conclusions

In this work, normally off HG-UTB HEMTs featuring a p-GaN/MIS-gate hybrid gate enabling flexible modulated *V*_TH_ are proposed. The p-GaN gate facilitates the enhancement of the depletion of the 2DEG channel, which further raises the energy band of the ultra-thin barrier heterojunction and results in a more positive *V*_TH_ in the proposed HG-UTB HEMTs. Moreover, the E-field crowing located at the p-GaN edge is effectively mitigated by the MIS-gate part, which is beneficial for reducing the gate leakage through the Schottky type p-GaN gate introduced by the high E-field. This work provides a simple approach to achieve normally off GaN HEMTs with promising device performance and particularly featuring flexible V_TH_ modulation ranges, which is of great interest for accommodating versatile power applications.

## Figures and Tables

**Table 1:** Key parameters of the HG-UTB HEMTs used in simulation.

Structural Parameters	Unit	Values
Source length (Ls)	μm	0.5
Drain length (Ld)	μm	0.5
Distance from source to gate (Lgs)	μm	1.0
Gate length (Lg)	μm	1.0
p-GaN length (Lp)	μm	0.5
MIS-gate length (Lm)	μm	0.5
Gate field plate length (Lfp)	μm	0.0 */1.0
Distance from gate to drain (Lgd)	μm	5.0
Thickness of p-GaN (Tp)	nm	70.0
Thickness of gate dielectric (Toxide)	nm	26.0
Thickness of passivation layer (TSiN)	nm	35.0
Thickness of GaN buffer layer (Tbuffer)	nm	2000.0
Thickness of transition layer (Ttrans)	nm	100.0
Al-mole fraction in AlGaN barrier (XAl)	/	0.18,0.20 *,0.23,0.25
Thickness of AlGaN barrier (*T*_AlGaN_)	nm	3,4,5 *,6,7
Hole concentration in p-GaN (*N*_p_)	cm^−3^	1,5,8,10 *,20,30,50 × 10^16^

* X_Al_ = 0.20, T_AlGaN_ = 5 nm, N_p_ = 1 × 10^17^ cm^−3^, L_fp_ = 0 µm are the default values.

## Data Availability

The data presented in this study are available on request from the corresponding author.
